# The Histone Deacetylase Inhibitor I13 Induces Differentiation of M2, M3 and M5 Subtypes of Acute Myeloid Leukemia Cells and Leukemic Stem-Like Cells

**DOI:** 10.3389/fonc.2022.855570

**Published:** 2022-04-12

**Authors:** Xiangyu Ma, Mengjie Zhao, Zhuo-Xun Wu, Jingfang Yao, Lei Zhang, Jinhong Wang, Zhenbo Hu, Liuya Wei, Zhe-Sheng Chen

**Affiliations:** ^1^ School of Pharmacy, Weifang Medical University, Weifang, China; ^2^ Department of Pharmaceutical Sciences, College of Pharmacy and Health Sciences, St. John’s University, New York, NY, United States; ^3^ Laboratory for Stem Cell and Regenerative Medicine, Affiliated Hospital of Weifang Medical University, Weifang, China

**Keywords:** acute myeloid leukemia, differentiation therapy, antigen processing and presentation, blockage of differentiation, HDAC inhibitor

## Abstract

Acute myeloid leukemia (AML) is a heterogeneous hematologic malignancy characterized by reduced differentiation of myeloid cells and uncontrolled cell proliferation. AML is prone to drug resistance and has a high recurrence rate during treatment with cytarabine-based chemotherapy. Our study aims to explore the cell differentiation effect of a potent histone deacetylase inhibitor (HDACi), I13, and its possible mechanism on AML cell lines (Kasumi-1, KG-1, MOLM-13 and NB4). It has been shown that I13 can significantly inhibit proliferation and colony formation of these AML cells by inducing cell differentiation coupled with cell-cycle exit at G0/G1. Mechanically, I13 presented the property of HDAC inhibition, as assessed by the acetylation of histone H3, which led to the differentiation of Kasumi-1 cells. In addition, the HDAC inhibition of I13 likely dictated the activation of the antigen processing and presentation pathway, which maybe has the potential to promote immune cells to recognize leukemic cells and respond directly against leukemic cells. These results indicated that I13 could induce differentiation of M3 and M5 subtypes of AML cells, M2 subtype AML cells with t(8;21) translocation and leukemic stem-like cells. Therefore, I13 could be an alternative compound which is able to overcome differentiation blocks in AML.

## Introduction

Acute myeloid leukemia (AML) is the most common clonal disease in adult acute leukemia and accounts for more deaths than any other leukemia (1). It is characterized by impaired differentiation of myeloid cells and aggregation of immature progenitor cells in the bone marrow. Acute promyelocytic leukemia (APL), the M3 subtype of AML, is one of the most aggressive forms and accounts for 10-15% of AML (2). For the treatment of APL, the combination of all-trans retinoic acid (ATRA) and arsenic trioxide (ATO) results in myeloid differentiation of the leukemic blasts and yields a 90% disease-free survival rate of 5 years. In addition, AML with (8;21) translocation is associated with 40-80% of M2 subtype of AML and 12-20% of AML in totality (3). Futhermore, AML with mixed-lineage leukemia (MLL) gene rearrangements (MLLr) associated with subtypes M4 and M5 of AML, is found in about 10% of AML patients with poor prognosis (4). Cytarabine (AraC) has been used as the cornerstone of induction therapy/consolidation therapy for non-APL AML. The 5-year survival rate of patients younger than 60 years of age with non-APL AML is about 40%, while only 10- 20% of those older than 60 years has achieved 5-year survival (5). The unsatisfactory outcomes of non-APL AML highlight the continuous need to develop novel therapies. Differentiation therapy with ATRA for APL has introduced a paradigm for success of cell differentiation therapy for AML (6). However, differentiation therapy with ATRA is not effective in non-APL AML.

Histone deacetylase (HDAC) is an epigenetic regulator of histone tail, chromatin conformation, protein-DNA interaction, and transcriptional and post-transcriptional modification (7, 8). HDAC inhibitors have been highlighted as a new category of anticancer drugs that regulate cell proliferation, differentiation and apoptosis through altering the acetylation status of histone and non-histone proteins (9, 10). Some HDAC inhibitors that have been approved in cancer therapy by the United States Food and Drug Administration (FDA) include suberoylanilide hydroxamic acid (SAHA), FK228, LBH58925 and PDX10124. SAHA (11) and FK228 (12) have been approved for the treatment of refractory cutaneous T-cell lymphoma. LBH58925 (13) and PDX10124 (14) have been approved for the treatment of multiple myeloma and peripheral T-cell lymphoma, respectively. This success has encouraged the developments of other HDAC inhibitors.

I13 is an indole-3-butyric acid containing HDAC inhibitor developed in our previous study (15). In the HDAC enzyme inhibitory assay, I13 exhibited IC50 value of 13.9, 49.9, 12.1 and 7.71 nM against HDAC1, 2, 3 and 6 comparing with SAHA (IC50 value of 50.7, 90.4, 164.1 and 169.5 nM, respectively). Moreover, I13 exhibited higher antitumor effects than SAHA in a HepG2 xenograft tumor model, likely by induction of apoptosis (15). In the present study, we evaluated whether I13 has significant activity against APL cells (M3 subtype of AML cells), M2 subtype of AML cells, especially t(8;21), M5 subtype of AML cells with MLLr and stem-like cells by inducing cell differentiation and elucidated the possible mechanism.

## Materials and Methods

### Chemicals

**Figure 1:**
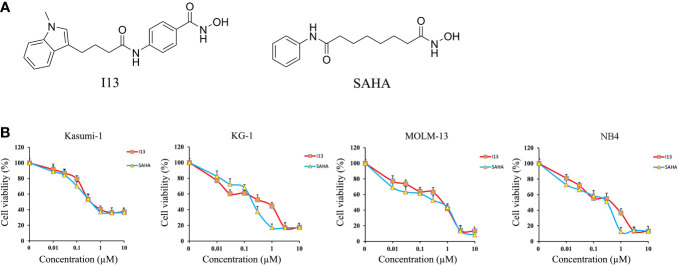
The anti-proliferation effect of I13 on Kasumi-1, KG-1, MOLM-13 and NB4 cells. **(A)** The chemical structure of I13 and SAHA. **(B)** The effect of I13 on the proliferation of AML cells (Kasumi-1, KG-1, MOLM-13 and NB4). Cells were treated with I13 or SAHA (0.01–10 µM) for 72 h and then evaluated by MTT assay. The points represent the mean and error bars represent standard error from three independent triplicate experiments.

I13 was prepared by our lab. The structure of I13 and SAHA is shown in **Figure 1A**. I13 and SAHA (10 mM) as stock solutions were dissolved in dimethyl sulfoxide (DMSO) and stored at -20°C. They were diluted with RPMI-1640 medium to the desired concentration. The same concentration of DMSO as that of the I13 solution was used as the control. The final concentrations of DMSO did not exceed 0.1% in all cultures, and they had no obvious toxic effect on cells. Fetal bovine serum (FBS) and RPMI-1640 were purchased from Sigma-Aldrich (St. Louis, MO, United States). The cell apoptosis assay was detected by staining solution containing propidium iodide (PI)/RNase and fluorescein isothiocyanate (FITC)-Annexin V (Becton Dickinson San Diego, CA, United States). MTT (3-(4,5-dimethylthiazol-2-yl)-2,5-diphenyltetrazolium bromide) and penicillin-streptomycin were purchased from Solarbio (Beijing, China). FITC anti-human CD11b (cat #301403), PE anti-CD13 (cat #301704 RRID: AB_314180), FITC anti-CD14 (cat #301804, RRID: AB_314186), and PE anti-CD15 (cat #301906, RRID: AB_314198) were obtained from Biolegend Inc. (San Diego, CA, United States). PE anti-human HLA-DR (Cat # FAB4869P) was obtained from R&D SYSTEMS (Minneapolis, MN, USA). Anti-human HLA-DP (Cat #A0906) was purchased from Santa Cruz Biotechnology (Santa Cruz, CA, USA). MethoCult H4100 (cat #04100) was obtained from STEMCELL Technologies (V ancouver, BC, Canada). Monoclonal antibodies against GAPDH (Cat #5174), H3 (Cat #4499), Acetyl-Histone H3 (Ac-H3, Cat #8173), CIITA (Cat #3793), and HLA-DRA (Cat #97971) were obtained from Cell Signaling Technology (Beverly, MA, United States). Monoclonal antibodies against HLA-B (Cat #DF7972 RRID: AB_2841362) were obtained from Affinity Biosciences (Affinity Biosciences, OH, USA).

### Cell Lines and Cell Culture

NB4 (M3 subtype of AML, APL cell line expressing PML-RARα, DSMZ: ACC 207), Kasumi-1 (M2 subtype of AML with t(8;21) translocation, DSMZ: ACC 220), MOLM-13 (M5 subtype of AML with MLLr, DSMZ: ACC 554) and KG-1 (leukemic stem-like cells established from the bone marrow cells of an AML patient, whose development was blocked at the myeloblast-promyelocyte stage of maturation, DSMZ: ACC 14) cell lines were used. All cells were maintained and cultured in RPMI 1640 medium containing 10% or 20% FBS and 1% penicillin-streptomycin. Cells were incubated at 37°C and 5% CO_2_ in a humidified incubator (Thermo scientific, Germany).

### Cell Proliferation Assay

MTT assay was performed to evaluate the effect of I13 on cell proliferation. Kasumi-1, KG-1, MOLM-13, and NB4 cell lines were cultured in 96-well plates at approximately 5,000 cells/well for 24 h. Then, the cells were incubated with I13 or SAHA (0.01-10 μM). After treatment for 72 h, 20 μL MTT (4 mg/mL) reagent was added to each well, and the supernatant was centrifuged after incubation for 4 h. Finally, 150 μL DMSO was added to each well, and the absorbance was measured at 570 nm by a microplate reader (Thermo scientific multiskan FC, Germany). The results were expressed as a percentage of cell viability standardized against DMSO-treated control cells.

### Colony-Formation Assay

About 5000 cells (Kasumi-1, KG-1, MOLM-13 and NB4 cells) were incubated with specific concentration of I13 in 500 mL of 2.6% methyl-cellulose medium (H4100) containing 10% FBS. After about 15 days, the number of colonies composed of more than 50 cells was calculated under an inverted microscope.

### Cell Cycle Analysis

Kasumi-1, KG-1, MOLM-13, and NB4 cells were incubated with varying concentrations of I13 (0.5 μM, 0.45 μM, 0.75 μM, and 0.5 μM, respectively) for 24, 48, or 72 h. These cells were washed with cold PBS and fixed with 70% ethanol at -20°C. Then, these cells were collected and stained with RNase A (100 mg/mL) and PI (50 mg/mL) at room temperature in the dark for 30 min. The percentage of cells in G0/G1, S and G2/M was determined by flow cytometry using the BD Accuri C6 plus flow cytometer (San Jose, CA, USA).

### Cell Apoptotic Rate Analysis

Kasumi-1, KG-1, MOLM-13 and NB4 cells were incubated with varying concentrations of I13 (0.25-1.5 μM) and SAHA (0.5-1 μM) for 72 h. Then, the cells were collected, washed with cold PBS, resuspended in 1× binding buffer and stained with FITC Annexin V/PI double labeling. After incubation for 30 min at room temperature in darkness, the apoptotic rate was quantitatively detected by flow cytometry with Cell Quest Software (FACSCalibur, BectonDickinson, United States).

### Analysis of Cell Morphology

To analyze cell morphology, Kasumi-1, KG-1, MOLM-13 and NB4 cells were incubated with I13 (0.5 μM, 0.45 μM, 0.75 μM, and 0.5 μM, respectively) for 72 h. The cells were centrifuged, dried naturally, stained with Wright-Giemsa for 10 min, washed with water, and dried. Finally, the slides were created and observed under light microscope.

### Analysis of Cell Surface Antigens

Kasumi-1, KG-1, MOLM-13 and NB4 cells were incubated with I13 (0.5 μM, 0.45 μM, 0.75 μM, and 0.5 μM, respectively). After 72 h, the cells were collected and incubated with antibodies at room temperature for 30 min in the dark. Finally, the cell surface antigens were detected by flow cytometry.

### Messenger Ribonucleic Acid (mRNA) Sequencing

Kasumi-1 was incubated with 0.5 μM of I13 for 48 h, and the cells were collected for RNA extraction and mRNA sequencing analysis. As described in our previous work (16, 17), the cDNA library was sequenced by Illumina genome analyzer. Levels of gene expression were estimated using fragments per kilobase of exon per million fragments mapped (FPKM) values. Differentially expressed genes (DEGs) were identified with a threshold (corrected p value 0.05 and log_2_ (folding change) ≥ 0.58). Gene Set Variation Analysis (GSVA) was conducted to analyze the potential signaling pathways involved. The clusterProfiler package of R software was used to perform the enrichment analysis of DEGs. The pathways with p value of < 0.05 were considered significantly enriched.

### Verification of Differentially Expressed Genes by Real-Time PCR Analysis

Kasumi-1 cells were incubated with 0.5 μM of I13 for 72 h. cDNA was synthesized using Primerscript RT reagent kit. Quantitative real-time PCR was performed on an Applied Biosystems 7500 Fast System (Thermo scientific, Germany) and the relative mRNA level of the target gene was measured *via* the 2−ΔΔCT method. Primers were used as follows: forward GAPDH: 5´-TGGGTGTGAACCATGAGAAGT-3´ and reverse GAPDH: 5´-TGAGTCCTTCCACGATACCAA-3´; forward CIITA: 5´- CCTGGAGCTTCTTAACAGCGA-3´ and reverse CIITA: 5´-TGTGTCGGGTTCTGAGTAGAG-3´; forward AML1-ETO: 5´- CACCTACCACAGAGCCATCAAA-3´ and reverse AML1-ETO: 5´- ATCCACAGGTGAAGTCTGGCATT-3´; forward HLA-DRA: 5´- AGTCCCTGTGCTAGGATTTTTCA-3´ and reverse HLA-DRA: 5´- ACATAAACTCGCCTGATTGGTC-3´; forward HLA-B: 5´- CGGAACACACAGATCTACAAGG-3´ and reverse HLA-B: 5´-GATGTAATCCTTGCCGTCGTAG-3´.

### Western Blotting Analysis

Cells were lysed with RIPA buffer containing protease inhibitors. The protein lysate was separated by sodium dodecyl sulfate polyacrylamide gel (SDS-PAGE) and transferred to PVDF membrane. After incubation with 10% skim milk for 90 minutes, the membranes were incubated with specific primary antibodies at 4°C for about 10 h, and then incubated with goat anti-rabbit immunoglobulin G (IgG) antibody at room temperature for 1 h. The protein expression was visualized using the enhanced chemiluminescence reagent detection system (FluorChem Q, Protein Simple, USA).

### Statistical Analysis

All data were expressed as the mean ± standard deviation (SD) of at least three repeated and independent experiments. One-way Anova was used for the comparison of each experiment group with the control using the SPSS software. p < 0.05 or p < 0.01 indicate statistical significance.

## Results

### I13 Significantly Inhibits Proliferation of Different Subtype of AML Cell Lines

MTT assay was used to detect the inhibitory effect of I13 on Kasumi-1, KG-1, MOLM-13 and NB4 cell lines. **Figure 1B** shows that I13 significantly inhibited the proliferation of Kasumi-1, KG-1, MOLM-13, and NB4 cells. The IC_50_ values for 72 h were 0.52, 0.51, 0.71 and 0.44 µM, respectively. These values were comparable to those of SAHA, which were 0.49, 0.24, 0.53 and 0.31 µM, respectively. This finding revealed that I13 has significant activity against the proliferation of M2, M3 and M5 subtype of AML cells including leukemic stem-like cells.

### I13 Remarkably Suppresses Colony-Formation Capacity of AML Cells

**Figure 2:**
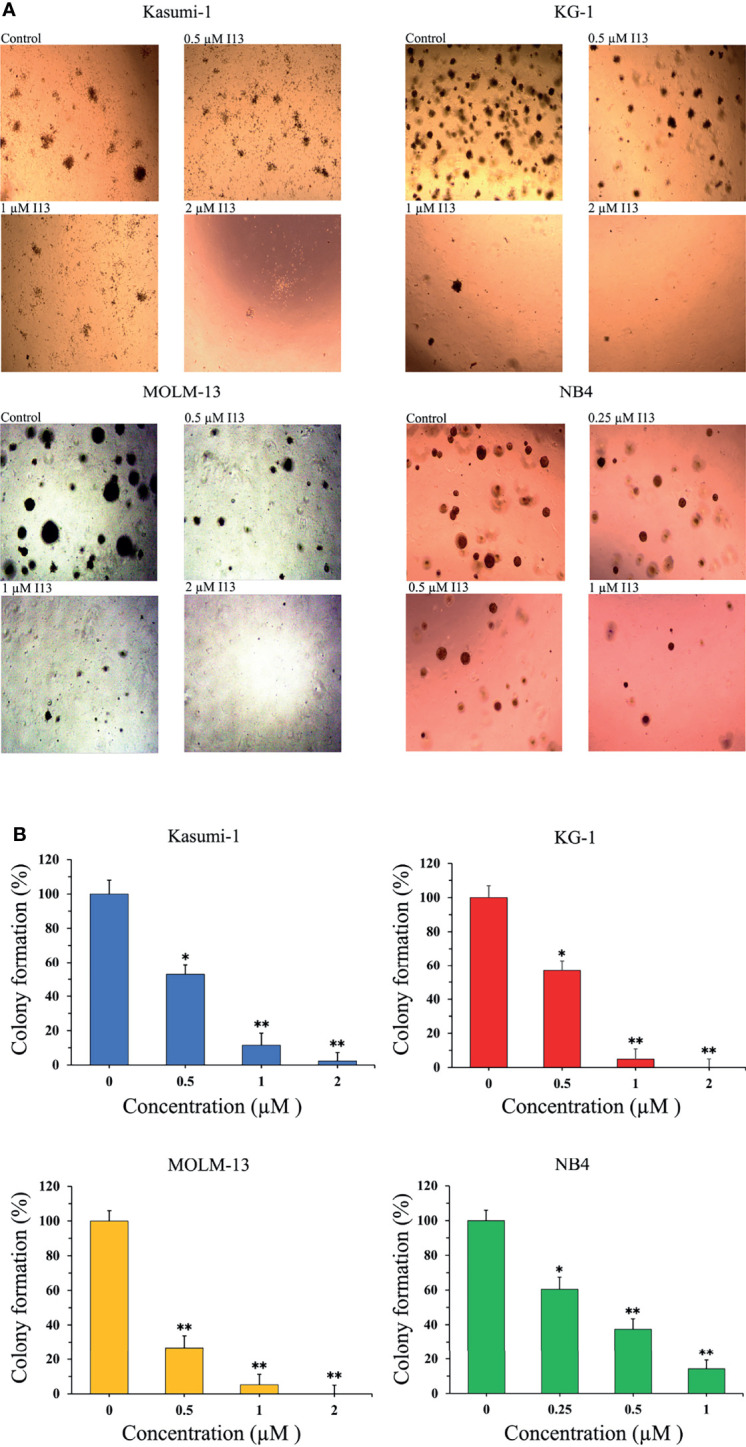
I13 reduced the colony-forming efficiency of Kasumi-1, KG-1, MOLM-13 and NB4 cells. **(A)** Kasumi-1, KG-1, MOLM-13 and NB4 cells were incubated with I13 (0.25–2 µM) for 15 days and examined with light microscopy. **(B)** A bar graph showing the statistical analysis of colony-formation number (*p < 0.05 and **p < 0.01). The analysis was performed three times.

The effect of I13 on the colony-formation capacity of Kasumi-1, KG-1, MOLM-13 and NB4 cells was next studied. As shown in **Figures 2A, B**, I13 (0.25-2 µM) remarkably suppressed colony formation capacity of Kasumi-1, KG-1, MOLM-13 and NB4 cells in a concentration-dependent manner. This result indicated that I13 could significantly inhibit the colony-formation capacity of M2, M3, M5 subtype of AML cells even leukemic stem-like cells at a low concentration.

### I13 Induces G1/G0 Arrest in AML Cell Lines

**Figure 3:**
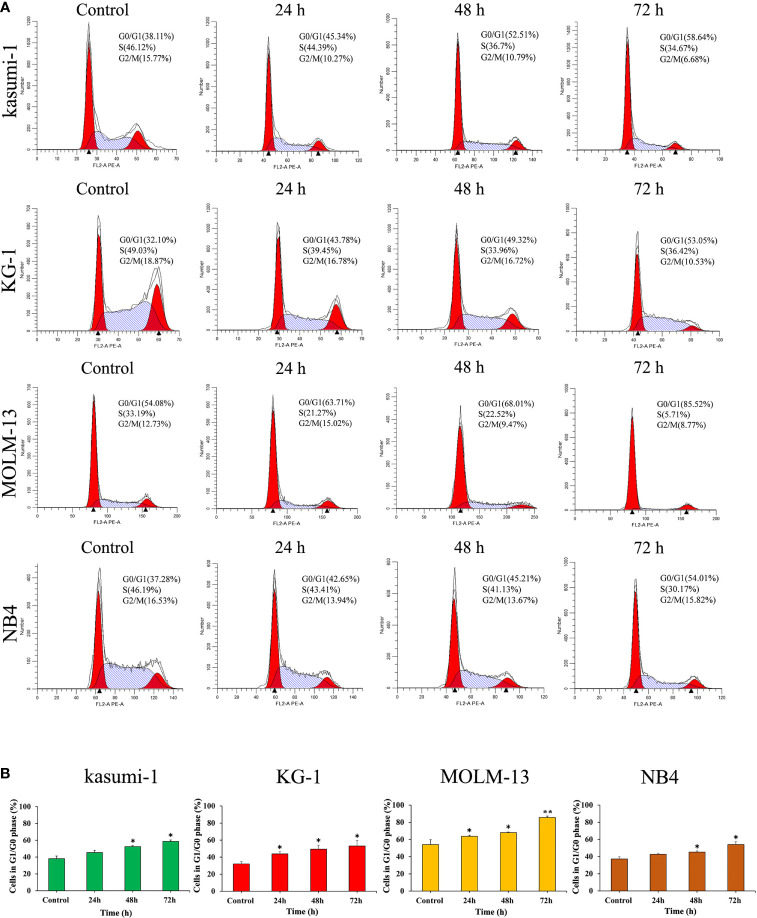
I13 induced the cell-cycle exit in Kasumi-1, KG-1, MOLM-13 and NB4 cells. **(A)** Kasumi-1, KG-1, MOLM-13 and NB4 cells were treated with I13 at concentrations of 0.5, 0.45, 0.75 or 0.5 µM, respectively, for 24, 48, or 72 h, then stained with PI, and detected by flow cytometry. **(B)** A bar graph showing the percentage of cells at G1/G0 phase (*p < 0.05 and **p < 0.01). The analysis was performed three times.

The effect of I13 on cell cycle distribution was evaluated in Kasumi-1, KG-1, MOLM-13 and NB4 cells. These cells were incubated with 0.5, 0.45, 0.75 and 0.5 μM I13, respectively for 24, 48 or 72 h. As shown in **Figures 3A, B**, the proportion of G0/G1 phase cells was significantly increased with increasing treatment time in these cells. These results indicated that I13 may induce G0/G1 arrest in these cells.

### I13 Induces Less Apoptosis in AML Cells

**Figure 4:**
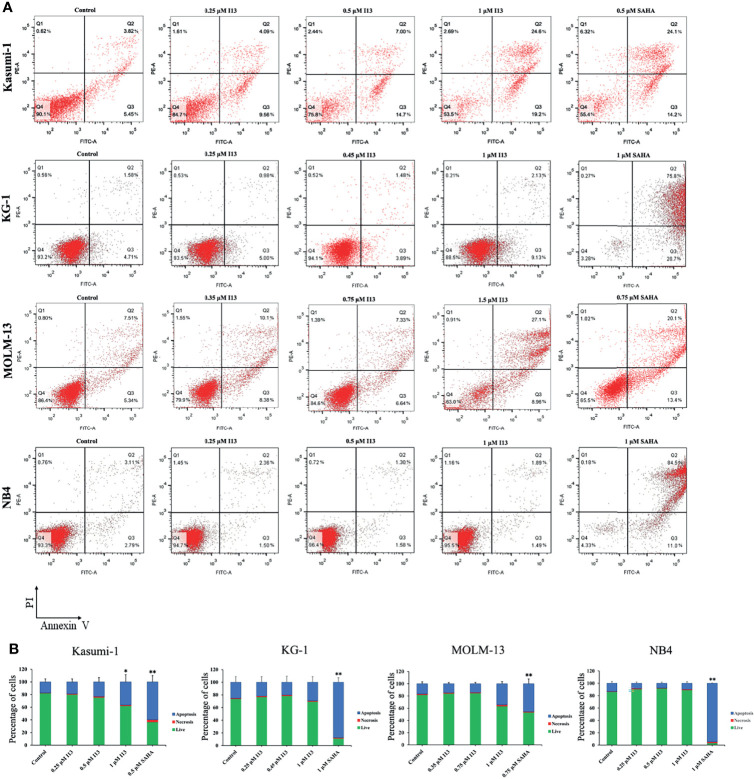
I13 reduced less apoptosis of Kasumi-1, KG-1, MOLM-13 and NB4 cells. **(A)** Kasumi-1, KG-1, MOLM-13 and NB4 cells were treated with I13 (0.25-1.5 µM) or SAHA (0.5, 0.75 or 1 µM) for 72 h. Cells were then stained with Annexin V-FITC/PI and detected by flow cytometry. **(B)** A bar graph showing the statistical analysis of apoptosis (*p < 0.05 and **p < 0.01). The analysis was carried out three times.

In order to determine whether the inhibitory effect of I13 is caused by the induction of apoptosis, Kasumi-1, KG-1, MOLM-13 and NB4 cells were treated with indicated concentration of I13 or SAHA for 72 h. It is shown in **Figures 4A, B** that no significant apoptosis was observed in Kasumi-1 and NB4 cells incubated with 0.5 μM I13. Similarly, less signs of apoptosis were found when KG-1 and MOLM-13 cells were treated with I13 under 1.0 and 1.5 μM, respectively. As a comparison, SAHA induced obvious apoptosis of these cells at the comparable concentration. This data suggests that the cell-cycle exit is not related to cell apoptosis in Kasumi-1, KG-1, MOLM-13 and NB4 cells treated with I13 at 0.5, 0.45, 0.75, 0.5 μM, respectively. Hence, these concentrations of I13 will be used to treat these cell lines in a subsequent experiment.

### I13 Promotes Differentiation in AML Cells

**Figure 5:**
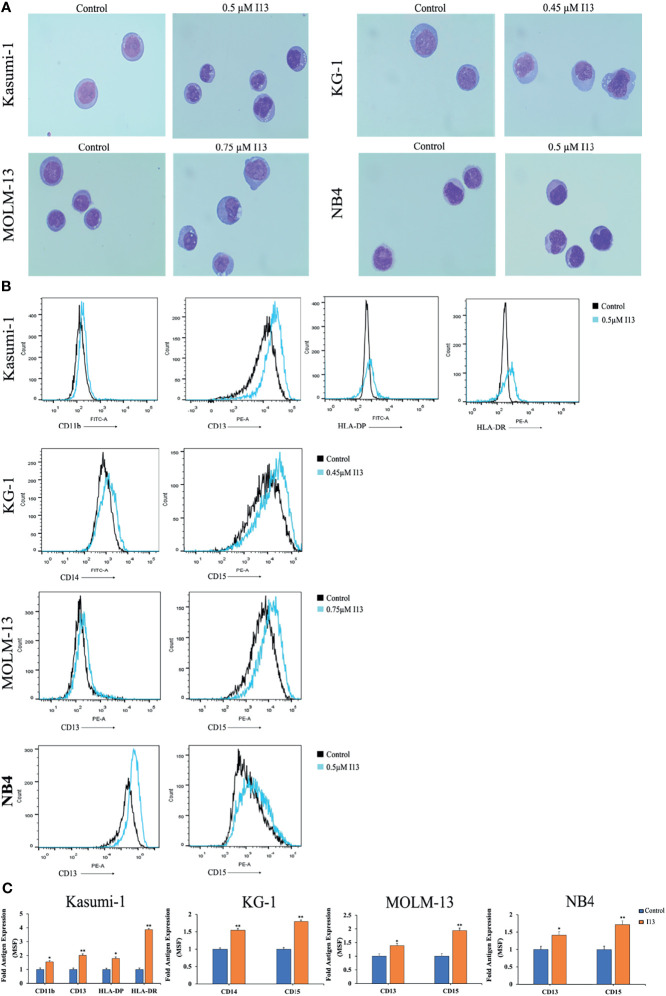
I13 induced cell differentiation of Kasumi-1, KG-1, MOLM-13 and NB4 cells. **(A)** Morphological changes of Kasumi-1, KG-1, MOLM-13 and NB4 cells captured by oil immersion lens (1000×). **(B, C)** The expression of cell surface antigens in Kasumi-1, KG-1, MOLM-13 and NB4 cells treated with 0.5, 0.45, or 0.75 µM I13 for 72 h. **(B)** Mean fluorescence intensity (MFI) of antigens. **(C)** A bar graph showing the statistical analysis of MFI (*p < 0.05 and **p < 0.01). The analysis was performed three times.

The above results indicated that I13 inhibits the proliferation of Kasumi-1, KG-1, MOLM-13 and NB4 cells, which is not associated with cell apoptosis. Hence, morphological and surface antigen analysis was performed to determine the differentiation of these cells treated with I13. It can be seen from **Figure 5A**, Kasumi-1, KG-1, MOLM-13 and NB4 cells undergo morphological changes with a decrease in the nuclear to cytoplasmic ratio and increased cell size. In addition, **Figures 5B, C** show that the expression of cell surface antigens CD14 and CD15 (markers of myeloid differentiation) were upregulated in KG-1 cells incubated with 0.45 μM I13. Similarly, 0.5 μM I13 induced differentiation of Kasumi-1 cells with an increasing expression of CD11b, CD13, HLA-DP, and HLA-DRA (major histocompatibility complex class II (MHCII)). In addition, 0.5 and 0.75 μM I13 also induced differentiation of MOLM-13 and NB4 cells, respectively, with increase in expression of CD13 and CD15. Therefore, the result suggests that the G0/G1 arrest of cell cycle induced by I13 may be caused by cell differentiation.

### I13 Induces Cell Differentiation Through HDAC inhibition Coupled With Exploiting the Antigen Processing and Presentation Signaling Pathway in Kasumi-1 Cells

**Figure 6:**
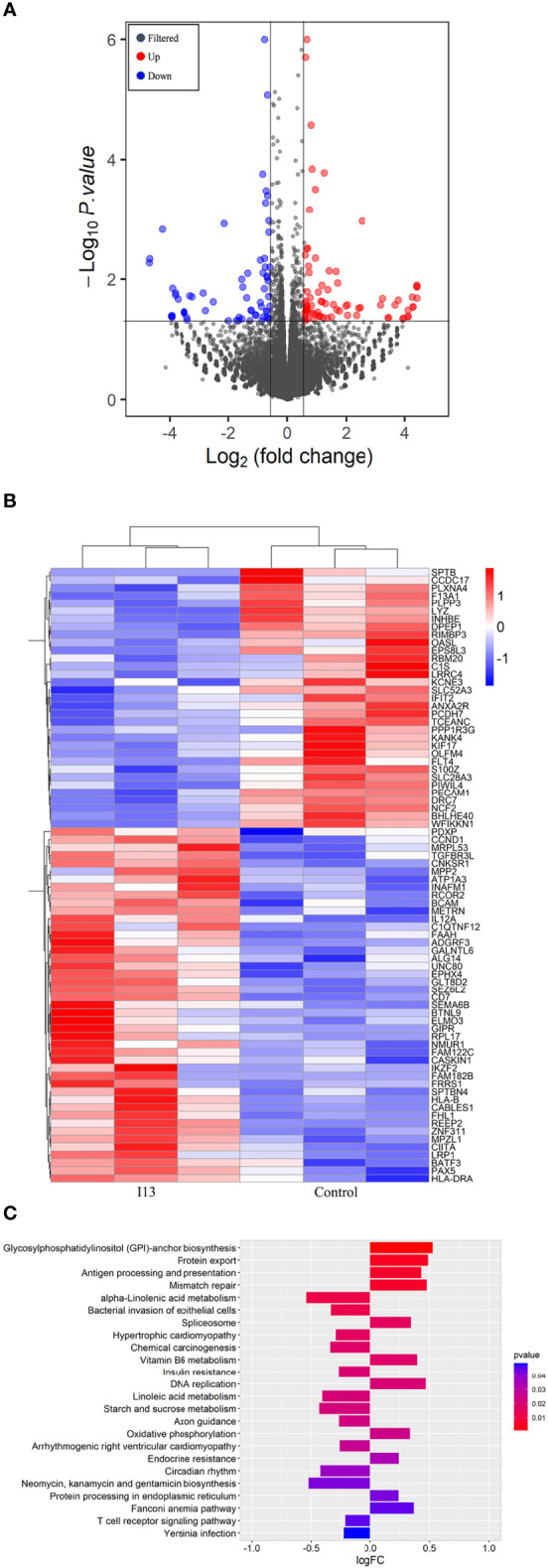
The cell differentiation effect of I13 is associated with antigen-processing and presentation-signaling pathways in Kasumi-1 cells. **(A)** Volcano plots of Kasumi-1 cells. **(B)** Heatmap of differentially expressed genes (p < 0.05 andlog2FC| > 0.58) according to their p-value. **(C)** The enriched pathways of differentially expressed genes using GSVA analysis. Kasumi-1 cells were incubated with 0.5 µM I13 for 48 h, and then mRNA sequencing was performed. The figures are representative of three independent experiments.

To understand the molecular mechanism involved in cell differentiation induced by I13, we performed an overall gene expression analysis of Kasumi-1 cells using mRNA sequencing. The volcanic diagram of Kasumi-1 cells is shown in **Figure 6A**. A total of 65 genes were downregulated and 76 genes were upregulated, indicating that I13 does not affect the mRNA expression of all genes universally. As shown in **Figure 6B**, CIITA, HLA-B, HLA-DP and HLA-DRA were significantly upregulated. These genes were enriched in the antigen processing and presentation signaling pathways by GSVA analysis **Figure 6C**.

**Figure 7:**
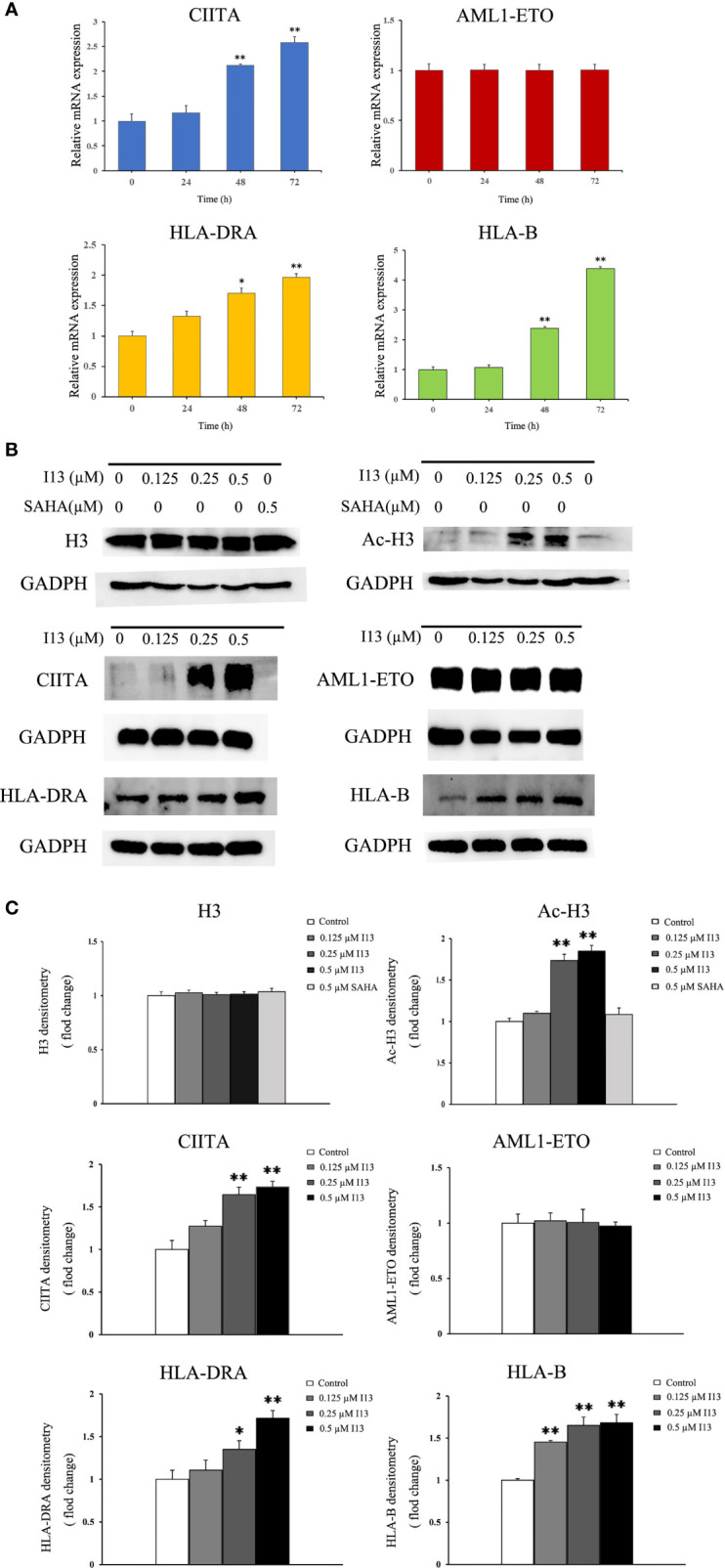
The expression of several genes and proteins related to cell differentiation or enriched in antigen-processing and presentation-signaling pathways in Kasumi-1 cells. **(A)** The mRNA expression of CIITA, AML1-ETO, HLA-DRA and HLA-B *via* real-time PCR **(B)** The protein expression of Ac-H3, H3, CIITA, AML1-ETO, HLA-DRA and HLA-B *via* Western blotting. **(C)** Protein expression was quantified by the software AI600 images. Kasumi-1 cells were treated with I13 (0.5 µM) for 72 h. *p < 0.05 and **p < 0.01. The analysis was carried out three times.

Since I13 is a HDAC inhibitor, we tested the HDAC inhibition by examining the level of acetylated histone protein H3 *via* Western blotting analysis. As shown in **Figures 7B, C**, Kasumi-1 cells with I13 treatment displayed significant concentration-dependent increase in the acetylated histone H3. Meanwhile, the same concentration of SAHA did not induce HDAC inhibition. In addition, the transcriptional and protein expression levels of CIITA, HLA-B, and HLA-DRA screened by mRNA sequencing were confirmed by RT-PCR and Western Blotting in Kasumi-1 cells. As shown in **Figures 7A–C**, I13 treatment significantly altered the mRNA and protein expression of CIITA, HLA-B and HLA-DRA. Because AML1-ETO fusion oncoprotein plays important roles in AML with t(8;21) translocation, the expression levels of AML1-ETO mRNA and protein were detected in Kasumi-1 cells. It is shown that I13 did not alter the transcriptional and protein levels of AML1-ETO.

## Discussion

AML, the most common type of acute leukemia in adults, is characterized by the proliferation of precursor myeloid cells with blocked differentiation (18). The use of Ara-C-based chemotherapeutic agents has been the dominant therapeutic approach. The prognosis has improved significantly in recent years, but remains poor particularly in elderly patients because of life-threatening toxicity of the medications (19). Moreover, relapses are still common, and long-term relapse-free survival is poor for most cases of AML leading to treatment failures. Hence, new therapeutic approaches are required. Differentiation therapy with ATRA for APL has dramatically improved the rate of complete remission and the long-term survival of APL patients (20). However, differentiation therapy with ATRA has not been used in non-APL AML. Therefore, the development of new compounds that can effectively induce cell differentiation in non-APL AML is a key area of investigation.

The Histone deacetylase inhibitor (HDACi), I13, has exhibited antitumor activity in xenograft models of human hepatocellular carcinoma by induction of apoptosis (15). In this study, we provided the first demonstration that I13 exhibited differentiation-inducing activity in AML cell lines. It has been shown that I13 could induce the differentiation of Kasumi-1, KG-1, MOLM-13, and NB4 cells, and inhibit cell proliferation and colony formation. Treatment with I13 resulted in G0/G1 phase arrest in these cells and cell line-specific morphological changes, and increased the expression of hematopoietic differentiation antigens, including HLA-B, HLADR, HLADP (MHCII, immune regulation antigens), and monocyte/granulocyte biomarkers such as CD11b, CD13, CD14 or CD15. Taken together, we found that I13 induces cell differentiation and is effective against M2, M3 and M5 subtypes of AML cells including leukemic stem-like cells.

HDACis have been clinically validated as a therapeutic strategy for the treatment of cancers or other diseases (21, 22). Moreover, HDACis have been shown to inhibit cell growth and proliferation through various mechanisms, including cell cycle arrest, induction of cell differentiation and apoptosis *via* the acetylation of histone (23–25). We therefore examined the level of H3 acetylation and found the accumulation of acetylated H3 in a concentration -dependent manner with the I13 treatment. In contrast, the same concentration of SAHA did not exhibit the HDAC inhibitory effect for no alteration of expression of Ac-H3, which suggested the activity of HDAC inhibition of I13 is stronger than that of SAHA in Kasumi-1 cells at comparative treatment concentration. These may suggest that I13 targeted the HDAC, and the effect of HDAC inhibition resulting in the acetylation of histone H3 contributed to the anti-proliferative activities by inducing cell differentiation.

In addition, T cell recognition antigens, also known as HLAs, are present in complex forms of MHC molecules on human cells (26, 27). HLAs play a pivotal role in the interaction of cancer cells with immune cells (28). In this study, we found that I13 could upregulate the expression of HLA-B (HLA class I antigen) in Kasumi-1 cells, indicating the possibility of immunity acquisition in differentiated Kasumi-1 cells. Furthermore, it is shown that the expression of HLA class II antigens are tightly regulated to ensure an immune response directed against malignant cells (29). In addition, evidence indicates that HLA class II antigen expression by tumor cells has a significant impact on their immunogenicity and the deletion of HLA class II eliminated donor T cells’ recognition of leukemia (30, 31). Based on these findings, we found that HLA-DRA and CIITA (the HLA trans-activator) were significantly upregulated in Kasumi-1 cells treated with I13. It was consistent with the upregulation of CIITA expression, which enhances the expression of HLA class II antigens (32). Therefore, it is suggested that increasing expression levels of HLA-DRA and HLA-DP may increase the acquisition of leukemic cells by immune cells, which potently exert helper function of immune cells. As mentioned above, CIITA, HLA-B, HLA-DP and HLA-DRA were enriched in the antigen processing and presentation signaling pathways. Therefore, I13 activated the antigen processing and presentation signaling pathways, indicating that I13 potently promotes immune cells to have a helper function or display an effector function with HLAs molecules in differentiated leukemic cells. Moreover, it is well known that antigen processing and presentation signaling pathway is a differentiation-related pathway, which denotes the development and differentiation of the hematopoietic cells into various cell types of hematopoietic lineages (33). Our present study showed that I13 treatment induced cell differentiation with morphological changes, increasing the expression of CD11b, CD13, HLA-DP and HLA-DR in Kasumi-1 cells. Further, it was known that cell fate involved in lineage commitment might be dictated by the activity of chromatin remodeling enzymes such as HDACs (34). Hence, the cell differentiation induced by I13 likely originate from the HDAC inhibition activity and subsequent activation of the antigen processing and presentation pathway in Kasumi-1 cells.

It was known that AML1-ETO generated by the t(8;21) translocation plays a central role in AML as a leukemia-promoting oncogene (35). The oncogene controls the leukemic phenotype in t(8;21)-carrying AML (36). We found that there was no significant change in the transcription and protein levels of AML1-ETO in Kasumi-1 cells. Therefore, the differentiation of AML cells induced by I13 was independent of the AML1-ETO gene.

Taken together, we found that I13 is effective against Kasumi-1 cells by inducing cell differentiation *via* presenting property of HDAC inhibition and exploiting the antigen processing and presentation signaling pathway.

In conclusion, I13 had significant anti-proliferative effect not only on M3 and M5 subtypes AML cell lines, but also on AML cells with t(8;21) translocation and leukemic stem-like cells. In addition, I13 can arrest the G0/G1 phase of these cells by inducing cell differentiation. The differentiation of kasumi-1 cells induced by I13 may be associated with the HDAC inhibition coupled with the activation of the antigen processing and presentation pathway. Moreover, I13 may have the potential to promote immune cells to exert a helper function or display an effector function. These findings reveal that I13 could be a potential lead compound for surmounting differentiation blockage in AML.

## Data Availability Statement

The datasets presented in this study can be found in online repositories. The names of the repository/repositories and accession number(s) can be found below: https://www.ncbi.nlm.nih.gov/geo; GSE193964.

## Author Contributions

XM: conceptualization, methodology, and writing-original draft. MZ, ZW, JY, XZ, LZ, and JW: methodology. ZH: supervision. LW: writing- editing and supervision. Z-SC: conceptualization and supervision. All authors contributed to the article and approved the submitted version.

## Funding

This work was supported by the National Natural Science Foundation of China (No 81700167 to LW), Science and technology support plan for youth innovation in universities of Shandong Province (2019KJM001 to LZ) and Natural Foundation of Shandong Province (Youth Found, ZR2019QH005 to LZ).

## Conflict of Interest

The authors declare that the research was conducted in the absence of any commercial or financial relationships that could be construed as a potential conflict of interest.

## Publisher’s Note

All claims expressed in this article are solely those of the authors and do not necessarily represent those of their affiliated organizations, or those of the publisher, the editors and the reviewers. Any product that may be evaluated in this article, or claim that may be made by its manufacturer, is not guaranteed or endorsed by the publisher.
